# Management of External Inflammatory Root Resorption following Tooth Autotransplantation Using a Modified Combination of Triple Antibiotics

**DOI:** 10.1155/2022/5178339

**Published:** 2022-06-15

**Authors:** Saeed Asgary, Ardavan Parhizkar

**Affiliations:** Iranian Centre for Endodontic Research, Research Institute for Dental Sciences, Shahid Beheshti University of Medical Sciences, Tehran 1983963113, Iran

## Abstract

External inflammatory root resorption (EIRR) is one of the most undesirable potential repercussions of various types of trauma and traumatic injuries to the tooth and its structure. This detrimental phenomenon may lead to severe complications, the consequent destruction of dental tissues, and eventual tooth loss. In the presented case, following the autogenous transplantation of tooth #17 as the host-tissue replacement for tooth #18, signs of EIRR were radiographically detected after 6 months. However, the thorough preparation of root canals, which involves complete cleaning and shaping, in addition to the application of a modified combination of triple antibiotics, consisting of penicillin G, metronidazole, and ciprofloxacin (PMC), managed to arrest EIRR. Moreover, the obturation of root canals using calcium-enriched mixture (CEM) cement as the endodontic biomaterial as well as the proper management of EIRR resulted in the healing of periradicular pathosis, abatement of clinical symptoms, and finally refunctionalisation of the tooth during a follow-up period of 2 years. It seems that the appropriate disinfection of the root canal system using PMC next to the prevention of microbial recontamination using CEM cement can perfectly manage EIRR.

## 1. Introduction

Resorption of dental hard tissues is shown to be associated with osteoclastic cell activity [1], an unwelcomed condition defined as the continuous loss of dentine and cementum owing to clastic cellular function [2], and could be considered the outcome of bacterial infection or chronic pulpal inflammation as the major predisposing factors [3]. External inflammatory root resorption (EIRR) is defined as a dynamic process which provokes the loss of different dental tissues, involving periodontal ligaments (PDL), dental hard tissues, and even the dental pulp in advanced stages [4]. Most often, the phenomenon initially reveals itself with small-sized lesions of cementum within PDL and, if not treated, may progress towards the involvement of the dental pulp [5]. Traumatic injuries, replantation of avulsed teeth, intentional (auto)replantation, and autogenous tooth transplantation are amongst factors that could initiate EIRR [6]. Since EIRR is a deteriorating lesion and could end in the gradual destruction of dental structure and subsequent tooth loss [7], it should be immediately diagnosed, properly managed, and perfectly treated. Consequently, the treatment of EIRR varies in accordance with the cause and severity of the corresponding resorption and its involvement with the pulpal tissue [8]. A recent study has claimed that regenerative endodontic procedures (REPs) can be elaborated as a potential solution for the treatment of EIRR; however, REPs are not contemplated as the first treatment option and need further clinical trials [9]. Nonetheless, it has been shown that immediate endodontic treatment and removal of microorganisms accompanied by the application of a suitable calcium silicate-based biomaterial could arrest and contrast the advancement of root resorption in reimplanted and avulsed teeth [6].

Furthermore, disinfection of the root canal system as well as maximum removal of intracanal microbiota seems to be a necessity in order to achieve successful outcomes in all endodontic treatments. To reach the best possible results, achieve the optimal removal, and attain plausible eradication of intracanal microorganisms, next to chemomechanical preparation of root canals, the application of intracanal medication has been endorsed [10, 11]. A collection of intracanal medicaments has been used for the disinfection of the root canal system, amongst which calcium hydroxide (CH) and triple antibiotic paste (TAP) have been extensively studied [12–15]. The latter has been recently used for REPs with varied degrees of success; however, it has been demonstrated that there are drawbacks to TAP, for example, crown discolouration and changes in dentine physical and mechanical properties [12]. Therefore, various modifications of TAP and antibiotics have been investigated for the removal of microorganisms from the radicular space [12, 16, 17]. A modified formula of triple antibiotics, which is a combination of penicillin G, metronidazole, and ciprofloxacin (PMC), has been recently studied, introduced, and used with successful results. This modified combination of triple antibiotics has been loaded on biopolymer-coated microparticles to combat endodontic microbiota. Seemingly, the novel PMC possesses a wide spectrum of antibacterial activity against dominant microspecies present in the radicular system [18].

Numerous studies have introduced calcium silicate-based cements, such as mineral trioxide aggregate (MTA), calcium-enriched mixture (CEM) cement, biodentine, and bioactive glass, as the commonest bioactive materials for use in endodontic applications [19–22]. CEM cement has been vastly used in different treatments since it has shown to exhibit favourable physical properties; reveal satisfactory biological properties; denote antimicrobial activity; have diverse clinical applications comparable to MTA, specifically in vital pulp therapy, repair, and sealing of perforation as well as resorption sites; and demonstrate similar sealing ability with MTA in the apical area and after root resection. Consequently, many investigations have recommended CEM cement to be used in different endodontic treatments, including, but not limited to, successfully sealing the apical portion of root canals and creating an apical barrier [23–25].

The prime objective of the current case is to report the management of severe EIRR due to autogenous transplantation of tooth #17 using PMC as the intracanal medication and CEM cement as the biomaterial for root canal obturation and root-end filling.

## 2. Case Report

A 36-year-old female patient was referred for consultation, possible evaluation, and probable retreatment of tooth #18 with an inappropriate previous root canal therapy and large periapical radiolucency shown around the mesial root in periapical radiography. The patient expressed severe pain as well as extreme discomfort in the mandibular left region during mastication and complained about a localised swelling around the abovementioned tooth.

**Figure 1:**
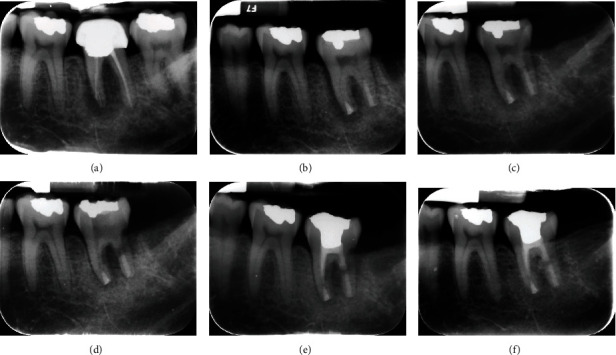
Mandibular left second molar with large periapical radiolucency: (a) mandibular left second molar with inappropriate previous root canal therapy, intracanal broken instruments, large periapical radiolucency, and furcation involvement; (b) former mandibular second molar was diagnosed hopeless and thus was removed whereas the mandibular left third molar was soundly extracted, and root-end cavities were cautiously prepared, carefully sealed using calcium enriched mixture (CEM) cement, and transplanted as a replacement for the mandibular left second molar; (c) in the 3-month follow-up, relative healing of periradicular bone was evident; however, radiolucencies in the mesial periapical region and furcation persisted, and signs of external inflammatory root resorption (EIRR) appeared on the distal root; (d) after the 6-month follow-up from the first treatment session and persistence of EIRR signs, the coronal restoration was removed, root canals were prepared, canals were medicated with a new combination of triple antibiotics (PMC), and the coronal cavity was sealed with Zonalin™; (e) after 3 weeks from the previous appointment, relative bone healing and complete arrest in EIRR progression were seen. Root canals were then filled with CEM cement, and the coronal cavity was restored using amalgam; (f) in the 2-year follow-up, thorough bone healing, disappearance of periapical and furcation radiolucency, and arrest/treatment of IERR were observed.

**Figure 2:**
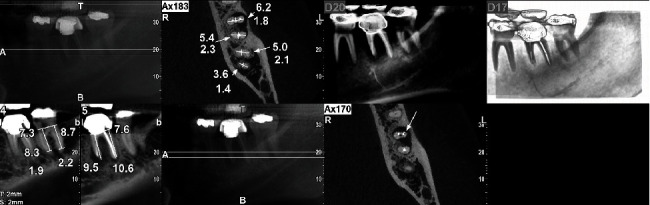
Cone-beam computed tomography (CBCT) showed large periradicular radiolucency around the mesial root in tooth #18, with sizable bone loss/resorption and the furcation involvement.

Initially, medical and dental histories were comprehensively taken, and extraoral as well as intraoral examinations were carefully conducted. In intraoral inspection, a prominent red-coloured swelling confined to a small area with no active pus discharge was seen on the left mandibular region, and tooth #18 was found to be highly sensitive to percussion. In mobility testing, mobility of grade III was seen, and in periodontal examination, the corresponding probing showed deep pockets (=9 mm) around the mesial root. Diagnostic radiographic evaluation of tooth #18 primarily showed broken instruments and large periapical radiolucency around the mesial root, involving the bifurcation area attributed to previous poor endodontic therapy (Figure 1(a)). In the same session, cone-beam computed tomography (CBCT) was obtained and confirmed large periapical radiolucency around the mesial root in tooth #18, severe bone loss, and rigorous furcation involvement of the tooth (Figure 2). Subsequently, due to the numerous problems and probable vertical root fracture, tooth #18 was considered hopeless; however, possible treatment options were broadly explained to the patient, comprising (i) simple extraction of tooth #18, with or without replacement using dental implants and (ii) simple extraction of tooth #18 with the autogenous transplantation of tooth #17. The patient strongly insisted on maintaining her own dental structure; as a result, and after the comprehensive explanation of possible procedures, the patient agreed upon the simple extraction of tooth #18 followed by the autogenous transplantation of tooth #17 as a replacement for tooth #18. Sequentially, informed consent was obtained from the patient.

In the first treatment session, with local anaesthesia in place using lidocaine with epinephrine 1 : 80,000 for the regional infra-alveolar nerve block, the sound extractions of teeth #18 and #17 were performed using cow-horn dental forceps for the detachment of the abovementioned teeth from their surrounding tissues in addition to their intact and minimally traumatic extractions. Tooth #18 was disposed; nevertheless, tooth #17 underwent needed preparation. To tailor the tooth for autotransplantation, the mesial and distal root ends of tooth #17 were cautiously resected, and small 3 mm deep cavities were created using a minipiezon ultrasonic retrotip (Joya electronics, Tehran, Iran). Root resection was carefully performed to create an appropriate matrix (the mentioned 3 mm cavities) so as to be filled and sealed with CEM cement and thus avoid further root canal treatment. Next, CEM cement (Bioniquedent, Tehran, Iran) was prepared via mixing its powder and liquid according to the manufacturer's instructions, and then, the root-end cavities were filled and sealed with CEM cement. Afterwards, tooth #17 was gently transplanted into the socket and stabilised using two figure 8 sutures in its mesial and distal aspects. After the radiographic confirmation of the procedure and the proper placement of the transplanted tooth, the patient was given verbal and written postsurgical instructions and dismissed for future follow-ups (Figure 1(b)). During the first month, the patient was recalled every week and examined clinically and radiographically. Intraoral exam showed no pathologic mobility in the transplanted tooth, the regional swelling had completely subsided, and the patient did not report pain under either mastication or percussion test. After 3 months, radiographic evaluation showed relative healing of the periapical bone around the mesial root; however, the radiolucency in the furcation area and around the distal root was still evident in spite of becoming smaller in size. Additionally, the closer radiographic evaluation showed signs of possible EIRR on the distal root (Figure 1(c)). The patient was dismissed for another period of 3 months. After 6-month recall from the 1^st^ treatment session, despite the healing of bone around the mesial root, the furcation and distal root seemed involved, and signs of EIRR appeared to progress; consequently, the coronal restoration was removed, proper access cavity was created, canal preparation (thorough cleaning and shaping) was performed, and the mesial as well as distal canals were medicated using PMC as the intracanal medicament to remove possible causative microbiota (Figure 1(d)). The tooth was temporised using Zonalin™ (reinforced zinc oxide eugenol) (Cina Bartar, Tehran, Iran), the patient was further recalled after 3 weeks, and the tooth was reevaluated clinically and radiographically clinically. In clinical examination, the tooth remained symptomless without any mobility or swelling, and in radiographic evaluation, progression in bone healing and relative limitation in EIRR was evident. Consecutively, the canals were filled with CEM cement, the tooth was restored with amalgam (Cina Faghihi, Tehran, Iran), and the patient was dismissed (Figure 1(e)). The arrest of EIRR was seemingly due to the proper preparation of root canals (cleaning and shaping of the root canal system), application of intracanal medication (PMC), and eventual filling of canals with the selected biomaterial (CEM cement). In a 2-year follow-up, the healing of bone surrounding the roots, arrest of EIRR, and normal PDL were observed (Figure 1(f)).

## 3. Discussion

In the present reported case, EIRR was caused by autogenous tooth transplantation for the replacement of a hopeless inappropriately endodontically treated tooth, whose initial signs were radiographically detected after 6 months. EIRR is reported to be the commonest type of external root resorption and may occur on various parts of the dental root. EIRR is classically defined as a resorptive lesion resulting from trauma, orthodontic treatment, or periodontal infection (microorganisms), causing an inflammatory response in PDL, leading to characterised deep resorptive defects, affecting and penetrating dentine as well as cementum, and triggering possible exposure of dentinal tubules [26]. The latter could contribute to bacterial invasion and the entry of microbial endotoxins into the dental pulp from PDL and vice versa and thus harsh impacts on the vitality and sensitivity of the tooth as well as PDL [27]. Therefore, when EIRR is diagnosed and radiographically observed, interceptive management of the malady consisting of the treatment of infected and pulpless root canal system is necessary; otherwise, EIRR could lead to the destruction of dental structure and eventual premature tooth loss if not properly diagnosed and immediately controlled or treated [28]. In addition to the spotted EIRR, the persistence of periradicular radiolucency was seen, showing possibly constant irritation (microorganism activity) in the region. Consequently, PMC was recruited as the intracanal medication to combat the effects of regional active microbiota, their by-products, and endotoxins. In the modified combination of triple antibiotics, penicillin G is used as a replacement for minocycline in the triple antibiotic paste (TAP) due to the unwelcomed impacts of minocycline on the physical and mechanical properties of dentine [29, 30]. PMC was introduced in 2020, where the triple antibiotics were experimentally employed to combat intracanal microorganisms, affecting gram-positive and several gram-negative bacteria, including *Enterococcus faecalis*, a dominant microorganism in endodontic failure [18]. Besides, the combination of penicillin G, ciprofloxacin, and metronidazole can create a white powder, which hypothetically may prevent tooth discolouration. Nevertheless, further studies are necessary to evaluate the possibility as well as prevention of tooth discolouration. In addition, when the signs of EIRR were radiographically revealed, the transplanted tooth received immediate root canal treatment; the existing coronal restoration was removed, the access cavity was carefully created, proper estimation of the remaining tooth structure was made, and the canals were skilfully filled using CEM cement biomaterial. CEM cement is a calcium silicate-based biomaterial which has shown biocompatibility, bioactivity, and anti-inflammatory properties alongside acceptable biointegrity and sealing ability. It has been applied as the root-end filling biomaterial with excellent results in various investigations for years [25, 31, 32]. Moreover, several in vitro studies have reported that CEM cement has demonstrated comparable properties to MTA, with notable outcomes in specific characteristics; for instance, CEM cement has experimentally shown to be a promising alternative for sealability in comparison to MTA [33–35]. When compared to MTA in vitro, lower microbial leakage properties in CEM cement could be related to its higher antibacterial activity [36], better marginal integrity, acceptable setting expansion, shorter setting time, and its bioactivity in saline environment to create hydroxyapatite-like crystals on its surface [37]. Furthermore, studies have disclosed that the bioactive CEM cement is capable of inducing hard tissues and encouraging osteogenesis and cementogenesis [38]. The long-term follow-ups in the reported case have shown proper healing of bone and resorption area, regeneration of large periapical lesions, and arrest of EIRR.

In the reported case, immediate root-end filling with CEM cement was performed and canals were medicated afterwards; however, the root canals were completely obturated following initial bone and periapical healing as well as the appearance of EIRR, causing successful outcomes. The deferral in the complete obturation of root canals follows the findings investigated by Shah and Logani and Jha et al., who have shown that the healing of periapical lesions could occur without immediate obturation of root canals, provided that the radicular system is flawlessly disinfected and completely sealed [39, 40]. Nonetheless, canal obturation favours the repair and healing of the periapical, periradicular, and surrounding tissues [41, 42].

The presented case report was conducted over a follow-up period of ~2 years. However, the lengths of follow-ups differ from one investigation to another. In his study, Abbott gave a period of 12 to 15 months for the treatment time when interceptive management of EIRR was taken into account [43]. Almost equally, Asgary and Fazlyab conducted their treatment of invasive cervical root resorption over a period of approximately 12 months using CEM cement as the endodontic biomaterial [44]. Di Giorgio et al. chose 24 months to perform their research on the arresting treatment of EIRR over reimplanted teeth using MTA [45], whilst Thomas believed it took a minimum of 4 weeks of rest for the initial repair process and 8 weeks of rest for the anatomic repair of EIRR to occur when the destructive phenomenon was caused by orthodontic forces [46]. Nonetheless, the effectiveness of applied treatment and the outcomes obtained can be better evaluated in longer follow-up periods [47].

## 4. Conclusions

The outcomes of the current case report showed a possible potential approach for the arrest and treatment of EIRR using a modified combination of triple antibiotics next to root canal therapy for the maximum removal of involved microorganisms and their by-products. Nevertheless, more clinical studies and detailed evaluations of conceivable long-term consequences or complications in addition to further well-designed clinical trials are mandatory.
